# 46733 Strategy for Effective Team Formation: A Case Study of Rutgers’ Big Ideas Initiative

**DOI:** 10.1017/cts.2021.684

**Published:** 2021-03-30

**Authors:** Ziyad Razeq, Biju Parekkadan, Nancy Reichman, Edmund Lattime

**Affiliations:** Rutgers University

## Abstract

ABSTRACT IMPACT: This study will provide valuable insight regarding the effectiveness of a top-down approach for team formation. OBJECTIVES/GOALS: Rutgers’ Big Ideas is a philanthropic initiative designed to gather team science ideas and present them to donors. We intend to evaluate this Team Science intervention and determine its feasibility in catalyzing the inception of team formation. We will explore the composition of teams that are formed using this particular method and team outcomes. METHODS/STUDY POPULATION: Our group will first evaluate the themes that were covered by the initial 210 submissions as well as the 40 ideas chosen to be presented at the Big Ideas Symposium. We will also be taking a look at the donor population that these ideas were presented to. Then, we will evaluate the 8-12 winning teams that were chosen to move forward. We will compare various success metrics of the 8-12 teams that were chosen compared to the 40 ideas that had not been chosen. RESULTS/ANTICIPATED RESULTS: Encouraging team science through an initiative such as the Big Ideas forum is not only feasible, but also highly effective in creating resilient teams that show prolonged productivity in fundraising, publications, and other academic metrics. DISCUSSION/SIGNIFICANCE OF FINDINGS: Team Science is an exciting movement with immense potential. To that extent, this study seeks to discuss ways that academic leadership can inspire and foster effective team science collaboration. Concurrently, our case review lays the groundwork for further improvements to Team Science initiatives.

